# The effect of NaOH on lowering interfacial tension of oil/alkylbenzene sulfonates solution

**DOI:** 10.1039/c7ra11287d

**Published:** 2018-02-07

**Authors:** Changming Zhao, Yulian Jiang, Mengwei Li, Tiexin Cheng, Wensheng Yang, Guangdong Zhou

**Affiliations:** College of Chemistry, Jilin University Changchun 130061 China zhougd@jlu.edu.cn

## Abstract

Six sodium *para*-dimethyl alkylbenzene sulfonates (PDABS, abbr. *p*-S12-5, *p*-S14-5, *p*-S16-5, *p*-S18-5, *p*-S16-6 and *p*-S16-8, respectively) have been synthesized. The structures and the purities of the products have also been confirmed by ^1^H-NMR and mass spectrometry. Crude oil and its equivalent alkanes were chosen as oil phases. The effect of the NaOH concentration on the interfacial tension (IFT) of PDABS was investigated by using a spinning drop tensiometer. The results showed that, in a certain range of NaOH concentrations, *p*-S14-5, *p*-S16-5 and *p*-S18-5 produced ultra-low IFT (∼10^−3^ mN m^−1^). NaOH was roughly similar to NaCl in aqueous solutions in terms of its effect on interfacial concentration and arrangement of PDABS at the interface. With the increase in the concentration of NaOH, the electric double layer of hydrophilic groups in PDABS was compressed to become smaller and the electrostatic interactions between hydrophilic groups weakened, which resulted in an increase in interfacial concentration of PDABS. Continuing to increase the concentration of NaOH, the electrical double layer was further compressed; water molecules could incorporate into the interface through loose hydrophilic groups. Therefore, these two aspects of variations caused IFT to display the trend of decreasing and then increasing with increase of NaOH concentration. However, at higher NaOH concentrations, PDABS molecules were driven into the oil phase by the salting out effect, and this process resulted in a decrease of IFT for water-soluble *p*-S12-5 and *p*-S14-5. For oil soluble *p*-S18-5, this process had little effect on the effective distribution in the oil phase. The effective distribution of PDABS in the oil phase played an important role in stabilizing the interface and reducing IFT. There was no clear evidence that NaOH reduced the IFT between oil and water by showing a synergistic effect between PDABS and active species formed *in situ* with acidic components in the crude oil.

## Abstract 2

The increase of NaOH concentration resulted in the compression of the electrical double layer. The distribution of PDABS in the oil phase improved the mutual attraction of lipophilic chains and the close-packed lipophilic groups at the interface.

## Graphical abstract

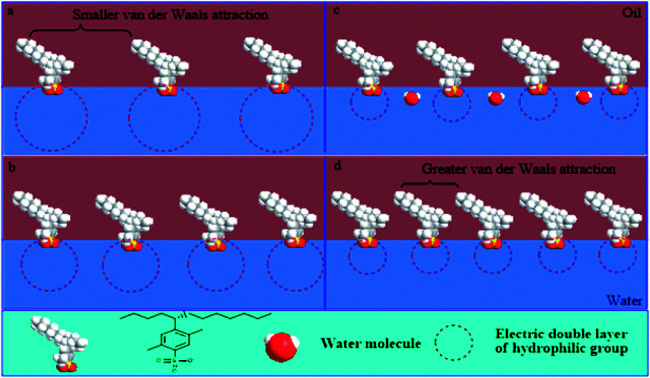

## 1. Introduction

In 1926, Harkins discovered the ultra-low interfacial phenomenon in the study of the interfacial tension (IFT) of the benzene/water system, induced by sodium oleic acid. However, due to the limitation of the method and the lack of urgent requirement for industrial production, this discovery caused little interest. In 1942, Vonnegut first proposed that in the absence of gravity, for the case of a lighter oil phase in a higher density phase at a known speed of rotation, a balance between the rotation centrifugal force and the IFT would be achieved.^1^ At this point the principle of minimum energy should be noted. Mathematically, an expression for IFT can be derived as a function of rotative angular velocity, diameter of the lighter phase and the density difference of the two phases. Based upon the above research, the spinning drop method for measuring ultra-low IFT (ULIFT) was realized for the first time. After this, researchers also discussed Vonnegut's theory in detail, improved the corresponding experimental equipment, and the relevant theories were verified by experiments.^2,3^ In these studies, the model proposed by Vonnegut was modified by different numerical expressions, to make it easier to adapt to different conditions and environments. To this day, the spinning drop tensiometer continues to be used to measure processes characterized by low IFT.

Theoretically, if the IFT can be reduced, the efficiency of chemical flooding will be greatly improved, which is the practical motivation for research into low IFT. Studies show that the displacement efficiency of surfactant chemical flooding is related to the capillary number *N*_c_, which will affect the saturation of the residual oil; the larger the *N*_c_ is, the higher the oil recovery efficiency is. Generation of ULIFT is one of the most effective ways to increase *N*_c_: only under ULIFT can a large amount of residual oil in porous rock be ejected.^4^ Alkyl benzene sulfonates (ABS) can produce ULIFT and are used as effective surfactants for chemical flooding.^5^ However, in actual production processes, due to the complex composition of industrial products,^6,7^ the lack of basic research on the interfacial performance of ABS and other factors results in some problems: for example, the actual field application of ABS has poor reproducibility and the site operations become complex.

The factors that affect the interfacial performance of ABS are complex and changeable. For example, the concentration of ABS itself, temperature, concentration of electrolytes in solution,^8–11^ organic bases,^12,13^ and so on can affect the reduction of IFT. Specifically, the ABS structure is the most complex factor. Doe *et al.*^14–16^ investigated the abilities of some surfactants to produce low IFT by synthesizing ABS with different numbers of alkyl substituents, and a number of significant results were obtained. Other studies have found that various components in the crude oil,^17–20^ as well as other organic additives,^21–23^ had an important impact on the interfacial performance.

It is generally considered that the acidic fraction in the crude oil undergoes reaction with the alkali to change the IFT of the oil/water phase. These fractions react with alkali (such as NaOH) in the aqueous solution to *in situ* generate surfactant–petroleum acid soap, which has a synergistic effect of greatly reducing IFT.^24–26^ In this paper, with *p*-xylene as starting material, several ABS homologues were synthesized carefully. The influence of NaOH concentration on IFT of the oil/water phase was determined by the spinning drop method, producing results of great significance for further clarifying the role of NaOH in reducing IFT of oil/ABS solutions.

## 2. Experimental section

### 2.1. Chemicals and instruments

All chemicals and reagents used are analytical grade. The purities and structures of products and intermediates were checked by NMR spectroscopy and mass spectrometry, respectively. IFT was measured by a TX-500C spinning drop tensiometer (produced by Shanghai Geological Research Institute).

### 2.2. Synthesis of sodium *para*-dimethyl alkyl benzene sulfonates

Straight chain fatty acyl chlorides with different alkyl groups R_1_, *p*-xylene and bromides containing different alkyl groups R_2_ were used as starting materials. By using Friedel–Crafts acylation, Grignard reaction, hydrogenation reduction, sulfonation and neutralization, six PDABS were synthesized. The synthetic route is as follows:
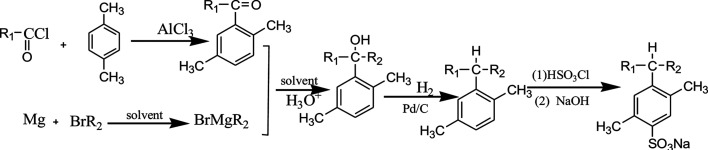


(*p*-S12-5: the 12 indicates that the alkyl chain has 12 carbon atoms; the -5 indicates that the aryl group is on the fifth carbon atom of the long alkyl chain; S: surfactant; *p*: *p*-xylene).

*p*-S12-5: R_1_ = C_7_H_15_, R_2_ = C_4_H_9_; *p*-S14-5: R_1_ = C_9_H_19_, R_2_ = C_4_H_9_; *p*-S16-5: R_1_ = C_11_H_23_, R_2_ = C_4_H_9_; *p*-S18-5: R_1_ = C_13_H_27_, R_2_ = C_4_H_9_; *p*-S16-6: R_1_ = C_5_H_11_, R_2_ = C_10_H_21_; *p*-S16-8: R_1_ = C_7_H_15_, R_2_ = C_8_H_17_.

First, a certain amount of anhydrous AlCl_3_ catalyst was added into a three-necked flask, followed by the addition of a certain amount of *p*-xylene. Then the flask was placed in broken ice under constant stirring, and straight chain fatty acyl chloride solution was slowly dropped through a constant pressure funnel for 1 hour. The temperature of the whole process was controlled below 10 °C under airtight and waterproof conditions. After dropping acyl chloride the reaction proceeded for 5 hours at 50 °C, yielding a red brown material (*p*-xylene : acyl chloride : aluminium muriate = 3 : 1 : 1.2, mole ratio). The red brown material was placed into a beaker filled with dilute hydrochloric acid and dissolved completely. NaHCO_3_ aqueous solution was added to obtain a neutral solution, which was then extracted with acetic ether. The supernatant liquid was washed several times with saturated NaCl aqueous solution, after which the solution was dried by adding anhydrous MgSO_4_. The desiccant was removed by filtering, and the acetic ether was removed by vacuum-rotary evaporation.

The unreacted *p*-xylene (boiling point: 138 °C) in the mixture was removed by atmospheric distillation. Finally, the alkyl aryl ketone was obtained by vacuum distillation.

Preparation of Grignard reagent requires that there is no water or oxygen in the solvent. Firstly, anhydrous ether was added into a three-neck flask filled with nitrogen, and the flask was joined to a reflux condenser; secondly, a few small pieces of sodium and a certain amount of diphenyl ketone as chromogenic agent were added under stirring at 50 °C. As the reaction proceeded, the color of the solution changed from colorless to dark blue gradually. Then, while stirring constantly, a small amount of anhydrous ether, magnesium chips (10 g, about 0.4 mol) and a few small pieces of iodine were added into the three-neck flask. Under the protection of N_2_, 3–5 ml of anhydrous ether and 0.2 ml of alkyl bromide were added into the above mixture in a proportion of 1 : 1 at 50 °C. After stopping heating, the mixed solution of ether and alkyl bromide are added into the above mixture continuously at room temperature, then heating to 80 °C and refluxing at 80 °C for 2 hours. After the reaction, the three-neck flask filled with Grignard reagent was put into an ice-water bath, and a certain amount of alkyl aryl ketone and anhydrous ether with mole ratio of 1 : 1 were also put into the flask to react for 2 hours at 50 °C. The mixed liquid was filtered with absorbent cotton, then extracted with acetic ether. The supernatant liquid was washed with saturated NaCl aqueous solution several times. The obtained solution was dried by anhydrous MgSO_4_ and the acetic ether was removed by vacuum-rotary evaporation. The alkyl bromide was removed by distillation under reduced pressure. The alkyl aryl tertiary alcohols were obtained finally.

Alkyl aryl tertiary alcohols, a certain volume of CH_3_COOH, HClO_4_ and a certain amount of 10% Pd/C were added into a high-pressure reaction kettle (PTFE lining) in turn. The air was completely evacuated from the reaction kettle. Hydrogen was pumped into the reactor at a pressure of 1 MPa. The reaction lasted for 2 hours at 50 °C with stirring. The reaction solution was filtered and desiccant MgSO_4_ was added. After 2 hours, the acetic acid was removed by vacuum-rotary evaporation, and the alkyl aromatics were obtained by decreasing the pressure.

The flask containing alkyl aromatics was put into an ice-water bath, at a temperature lower than 15 °C. Then, 3 g HSO_3_Cl (105 wt% of alkyl aromatics) was added by dropping, and the product in the flask turned into a red brown viscous liquid. After slowly adding anhydrous ether, NaOH aqueous solution with a concentration of 20% was added to adjust the pH value of the solution to 10. The obtained product was crude sodium *para*-dimethyl alkyl benzene sulfonate. The unsulfonated alkane was removed from the product by adding petroleum ether. Then, the remaining content of the flask was continuously extracted with ethanol–water solution (volume ratio = 2 : 1), and the target product was obtained by recrystallization.

### 2.3. Measurement of interfacial tension

The IFT between oil and aqueous phases was measured by the spinning drop method. First of all, a series of PDABS aqueous solutions with different NaCl and (or) NaOH concentrations were prepared, and the solutions were placed in a thermostat water bath at 45 °C. A TX-500C IFT tensiometer was used at 45 °C, and the rotation speed was 7000 rpm. The measurement tube was cleaned by acetone several times and rinsed by the PDABS solution. A tube containing PDABS solution and an alkane oil phase (1 μL) was put in the instrument to measure IFT. The formula for calculation of the specific IFT is given below:
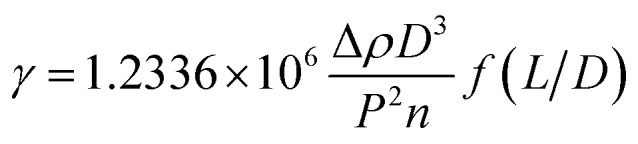
*γ* – interfacial tension (mN m^−1^), Δ*ρ* – density difference between oil and water phases (g cm^−3^), *L* – length of oil drop (cm), *D* – diameter of oil drop (cm), *P* – reciprocal rotation rate (ms·per rev), *n* – refractive index of aqueous phase (generally *n* = 1.330), *f*(*L*/*D*) – correction factor (when *L*/*D* ≥ 4, *f*(*L*/*D*) = 1).

## 3. Results and discussion

### 3.1. The *n*_min_ values of different surfactant solutions and equivalent alkane carbon number (EACN) of crude oil

In a study on IFT between octane, octyl benzene or butyl cyclohexane and a particular equivalent weight surfactant with a given electrolyte concentration, Cayias *et al.*^27,28^ found that appropriate mixtures of other hydrocarbon members of these series will produce low IFT if the average molecular weight is identical to octane. That means, for a complex mixture of alkanes or crude oil, normal paraffin can be used for researching IFT properties. Practice has proved that this is an effective method to study the IFT behavior of ABS during chemical flooding. At the same time, the concept of the minimum alkane carbon number (*n*_min_) has been proposed, and the *n*_min_ reflects whether a minimum IFT may be produced between the surfactant solution and oil phase. According to the *n*_min_, Cayias proposed the concept of equivalent alkane carbon number (EACN) for any hydrocarbon, hydrocarbon mixture or crude oil when studying IFT. A series of mixture solutions with different equivalents can be obtained by mixing homologues of the surfactant; in other words, the *n*_min_ value in each of the surfactant solutions with different equivalents is different, and these *n*_min_ values are varied as far as possible across the range of alkane carbon number. Then the IFT is measured between the crude oil and every surfactant solution with different equivalents. A plot of IFT *versus n*_min_ is drawn. The minimum value of *n*_min_ in the curve is the EACN of the crude oil.

**Fig. 1:**
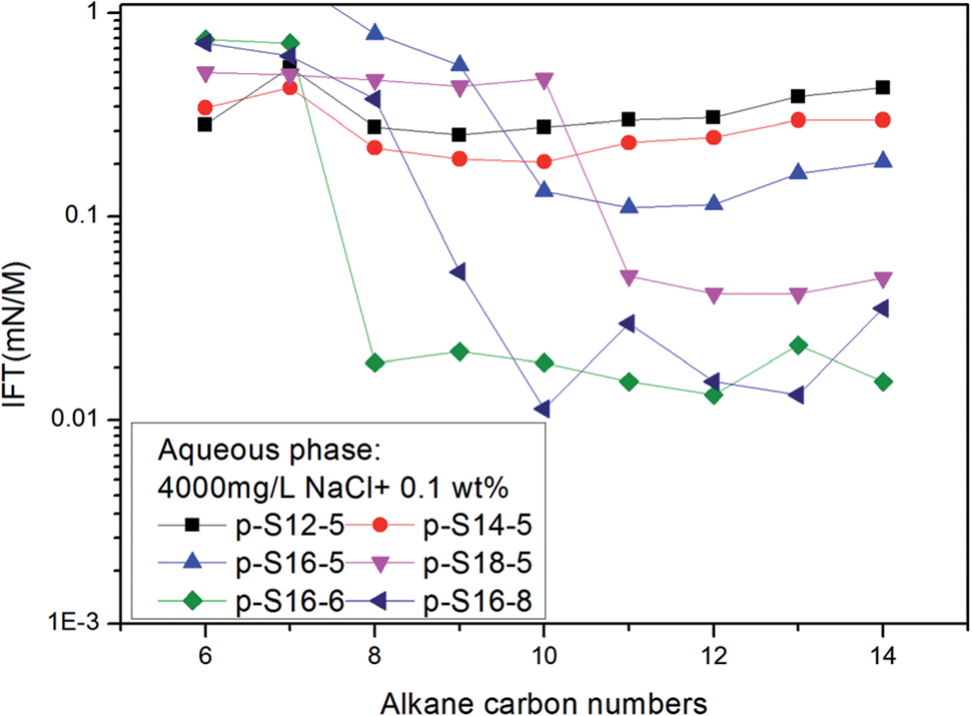
The variation of IFT for six surfactant aqueous solutions against a series of normal paraffins.

The *n*_min_ values of the six PDABS aqueous solutions are shown in Fig. 1. It can be seen that the *n*_min_ values are a function of alkyl chain length, *i.e. n*_min_ increases with increasing length of the long chains. This trend breaks down for *p*-S16-6 and *p*-S16-8, which indicates how sensitive the *n*_min_ values are to the position of the ring on the chain.

**Fig. 2:**
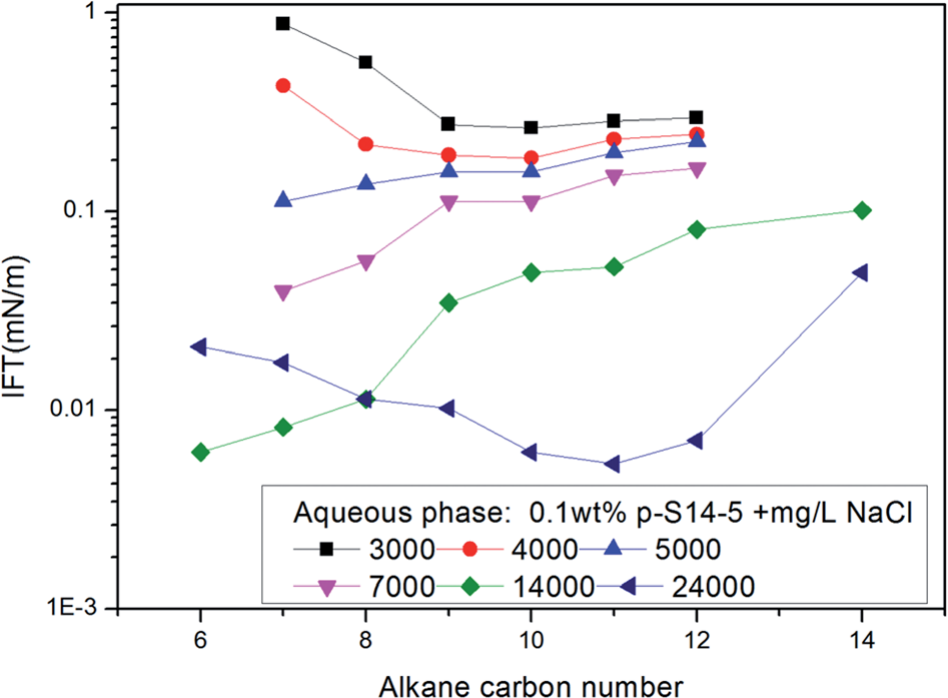
Effect of the NaCl concentration on *n*_min_ of *p*-S14-5 aqueous solution.

**Fig. 3:**
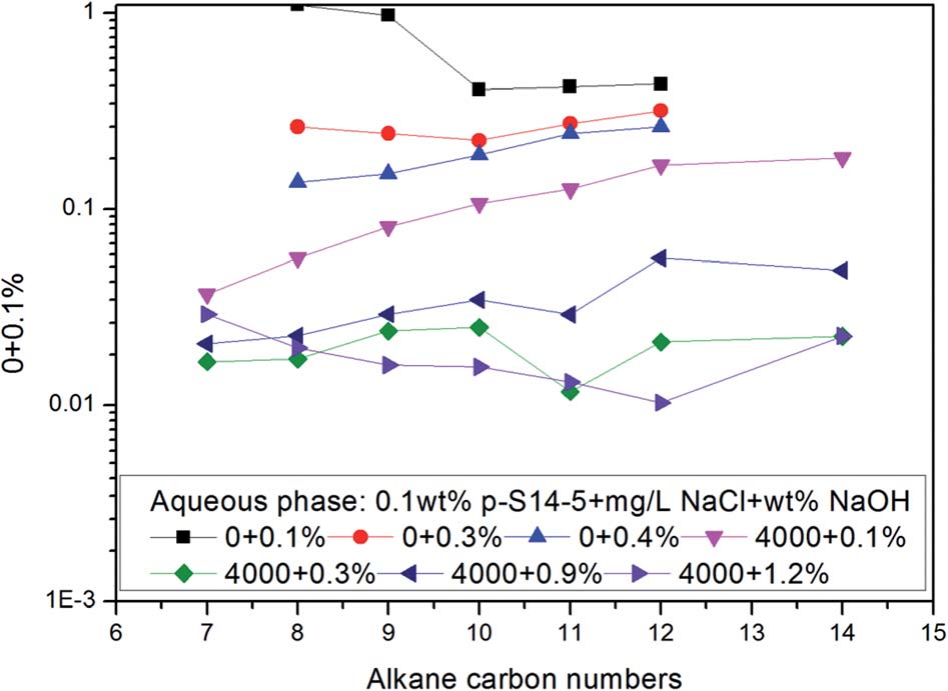
Effect of the NaOH concentration on *n*_min_ of *p*-S14-5 aqueous solution.

The effects of NaCl and NaOH concentration on the *n*_min_ values are illustrated in Fig. 2 and 3 respectively. We can see from Fig. 2 and 3 that the *n*_min_ values increase with increasing NaCl and NaOH concentration. The IFT decreases with increasing NaCl concentration, but for NaOH, the IFT increases again at the concentration of 0.9 wt% NaOH.

**Fig. 4:**
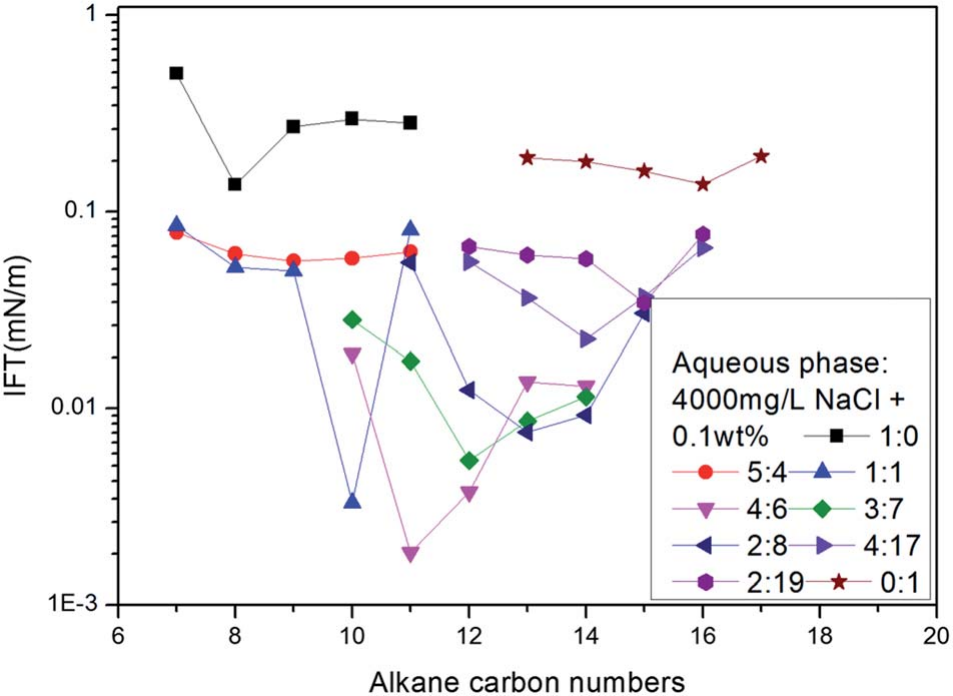
The variation of IFT for mixed solutions with different ratios of 12-5 and 16-8 against a series of normal paraffins.

Following the literature,^17^ sodium 2-methyl-5-(5-dodecyl)-benzene sulfonate (12-5) and 2-methyl-5-(8-hexadecyl)-benzene sulfonate (16-8) were selected to perform an alkane scan. The *n*_min_ values of several binary mixture solutions were measured for the series between octane and hexadecane, as shown in Fig. 4. The curve showed that the *n*_min_ values increased with the increase of average molecular weight of surfactant mixtures under given conditions.

**Fig. 5:**
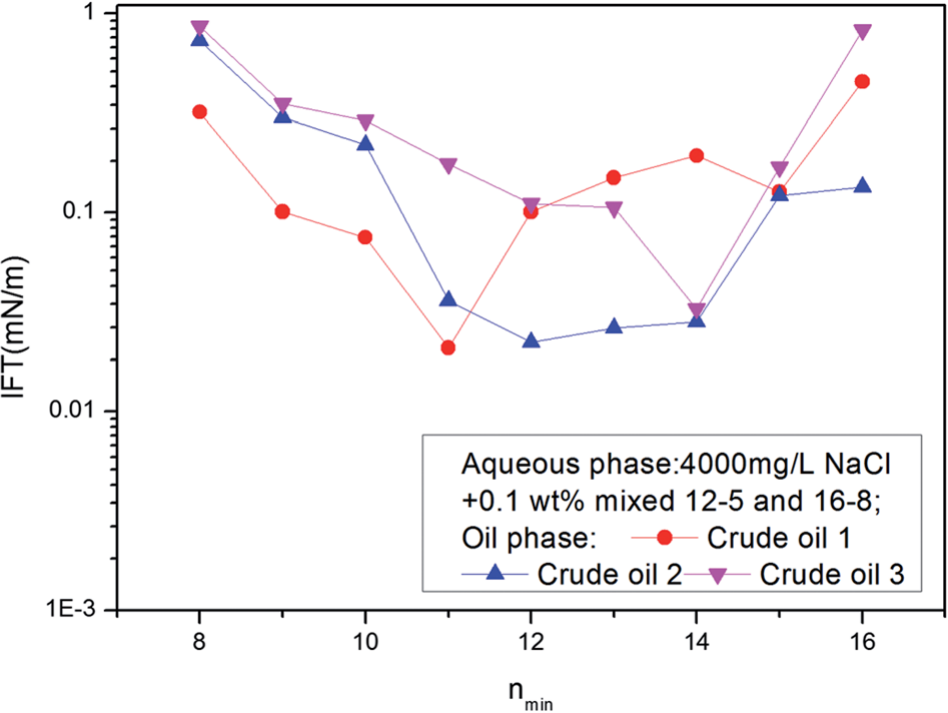
The IFTs between surfactant solutions created by mixing 12-5 and 16-8 in different ratios and crude oils 1, 2 and 3.

The IFT between these ABS mixture aqueous solutions and different crude oils was measured. A curve was drawn with *n*_min_ as transverse coordinate and IFT as vertical coordinate. If the curve has a minimum, the *n*_min_ value of that point defines the EACN of the crude oil. The above mixtures were used for the determination of the EACN of different crude oils in Daqing oil field. The results are shown in Fig. 5. The EACN for crude oil 1 was 11; and those for crude oil 2 and 3 were 12 and 14 respectively.

The EACN value is an invariant property of crude oil, in other words, it does not depend on factors such as surfactant structure *etc.*^14^ For a surfactant, it is easier to distribute into crude oil with lower EACN. If the EACN value changes, the characteristics of the oil phase may change correspondingly. Therefore, the properties of crude oil can be changed by adding other components to change the EACN value of the crude oil. Zhang *et al.*^29,30^ added carboxylic acids with different chain lengths or *n*-octadecanol to change the EACN value of a model oil. They found that a minimum interfacial tension (IFT_min_) is easier to generate if the ratio of the EACN value of the model oil to the *n*_min_ of the surfactant is closer to one. Otherwise, when the ratio is far from one, the IFT_min_ cannot be obtained so easily.

The effect of NaOH concentration in aqueous solution on lowering IFT was investigated herein by using crude oil and corresponding equivalent alkanes as the oil phase after measuring the EACN value of the three kinds of Daqing crude oil.

### 3.2. Effect of NaOH concentration on interfacial tension when using normal paraffin as oil phase

Regarding the role of NaOH in ASP (alkali/surfactant/polymer) flooding, it is generally believed that NaOH can *in situ* generate surface active substances by reaction with acidic material in crude oil. In turn, the synergistic effect between surfactant and surface active substances can effectively reduce the IFT between oil and water.^24–26,29,30^ When using normal paraffin as a model oil phase, the oil phase does not contain any acid that can react with the alkali to generate a surface active substance. Thus the added NaOH cannot interact with the acid composition, colloids and bitumen in crude oil, and therefore cannot *in situ* form surface active substances to effectively lower the IFT of the oil/water phase as described in the literature.

In systems of normal paraffin and ABS, NaOH is used as the electrolyte to change the ionic strength of the aqueous phase, and thus, to change the structure and size of the electrical double layer of the polar group of ABS, in turn affecting IFT. Miller and Scriven^31^ have pointed out that the interaction of the electrical double layer close to the interface can cause instability of the interface, thus the chemical potential and interfacial free energy of the interface can be changed, which would strongly influence the ability to obtain low IFT.

The interface between oil and water is known to be a relatively stable dynamic system.^4^ When the IFT is stable, the interfacial concentration of surfactant is in a dynamic equilibrium. Therefore, a slight disturbance of the interface can affect the stability of the interface, which causes the fluctuation of IFT. Therefore, the high speed spinning drop method was used herein to test the IFT between the oil and water phases, in order to obtain a relatively stable instantaneous value of IFT.

One of the factors lowering the IFT is known to be the increase of the interfacial concentration of surfactant. The interfacial concentration is related to the interfacial area occupied by the surfactant molecules; the smaller the effective cross-sectional area of the surfactant at the interface is, the greater its interfacial concentration is. Therefore, interfacial concentration depends on the structural groups in the surfactant molecules and their arrangement at the interface. In general, for surfactants with a single hydrophilic group, the area occupied by a surfactant molecule at the surface is mainly determined by the size of the electric double layer of the hydrophilic groups.

**Fig. 6:**
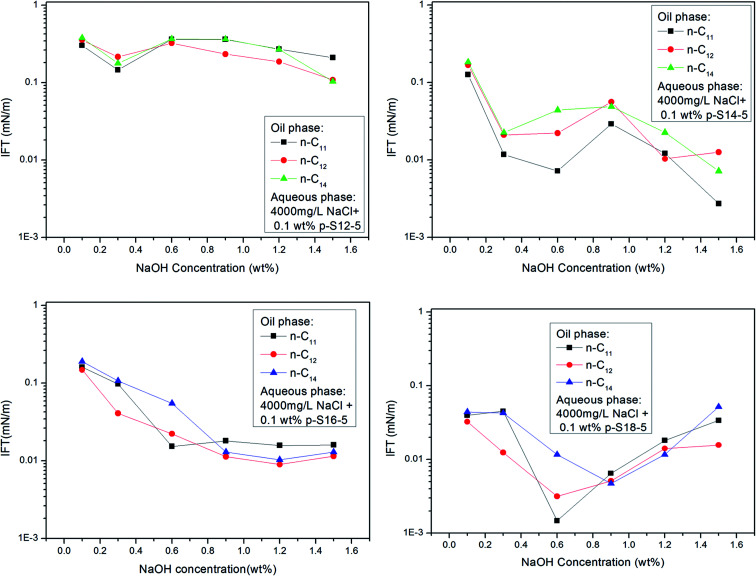
Effect of NaOH concentration on IFT of PDABS homologues.

Fig. 6 shows that the IFT of *p*-S12-5, *p*-S14-5, *p*-S16-5 and *p*-S18-5 homologues (in which the benzene ring is linked to the fifth carbon atom) changes as a function of NaOH concentration. For *p*-S12-5 and *p*-S14-5 aqueous solutions, the IFT of oil/water decreases, then goes up, and finally decreases again with the increase of NaOH concentration. At low electrolyte concentration, the interfacial concentration of PDABS is lower, which leads to a higher IFT. The appearance of a low IFT value can be attributed to the increase of NaOH concentration, the compression of the electric double layer, the decrease of electrostatic repulsion and the increase of interfacial concentration of PDABS, so a low IFT value will be produced at the appropriate NaOH concentration. Under continuous increase of the NaOH concentration, it is therefore to be expected that in the presence of a higher concentration of electrolyte, the electrical effects are shorter ranged than in a lower concentration of electrolyte, that is, the electrical double layer is further compressed. This will cause the polar water molecules to incorporate into the interface through loose hydrophilic groups. This, in turn, causes the rise of IFT.

During further increase of NaOH concentration, the water-soluble PDABS will be transferred into the oil phase by the “salting out” effect. Under intensification of this effect, the interfacial concentration of PDABS will increase and interface will become stable, thus IFT will be reduced. The higher NaOH concentration produces an increase in the interfacial concentration and stability of the interface; this is probably the result of mutual attraction of lipophilic chains and the close-packed lipophilic groups at the interface resulting from the effective distribution of PDABS in the oil phase. When the mutual attraction of the lipophilic groups is sufficient to overcome the mutual repulsion of the hydrophilic groups, then close-packing of the lipophilic chains may occur.

In discussing the effect of electrolyte concentration on IFT, Chan and Shah elaborated the effect of the “salting out” process on the concentration of the active substance at the interface and on the reduction of IFT.^4^ They argued that surfactant molecules can enter into the oil phase by the “salting out” process with the increase of salt concentration. Under a suitable salt concentration, surfactant molecules will re-distribute between the oil and water phases until equal concentrations are reached. At this time, the affinities between surfactant molecules at the interface and between the two phases of oil and water are also equal. This fact leads to a high interfacial concentration and, thus, a low IFT value. However, during measurement of the IFT by the spinning drop method, the volumes of the oil and water phases are significantly different. Therefore, the concentration of the surfactant in the aqueous phase should be constant. It is questionable whether equal concentrations of the two phases can appear and the maximum interfacial concentration can be generated. Even so, the minimum interfacial tension can be generated under the largest interfacial concentration and the surfactant can distribute into the oil phase by the “salting out” effect. Both of these two viewpoints are useful for reference.

Because *p*-S16-5 and *p*-S18-5 have long alkyl chains, their solubilities in the oil phase are higher than that of *p*-S12-5 and *p*-S14-5. It can be seen from Fig. 6 that the IFT decreased to a low value and then increased with the increase of NaOH concentration. This means that for an oil-soluble surfactant, the increase in NaOH concentration only changes the ionic strength of the aqueous phase, so it influences the structure of the electrical double layer and the electrostatic interaction. This has little effect on the distribution of oil-soluble *p*-S16-5 and *p*-S18-5 in the oil phase. Thus, the IFT decreases and then increases with the increase of NaOH concentration.

Fig. 6 shows that for *p*-S18-5, with a longer lipophilic alkyl chain, the IFT is not decrease by the salting out effect at higher concentration of NaOH. This is evidence that the ionic strength of the aqueous phase has little effect on the distribution of oil-soluble PDABS in the oil phase. Meanwhile, it is important that effective distribution of ABS surfactants in the oil and water phases can lower IFT, and maintain the stability of IFT. Chan and Shah showed the importance of an effective distribution of ABS surfactants in the oil and water phases to lower IFT.^4^ Only with an effective distribution of surfactant in the oil and water phases can ULIFT be reached. Therefore, the distribution of surfactant in the oil phase also has a major impact on the ability of the interface to maintain a low IFT.

**Fig. 7:**
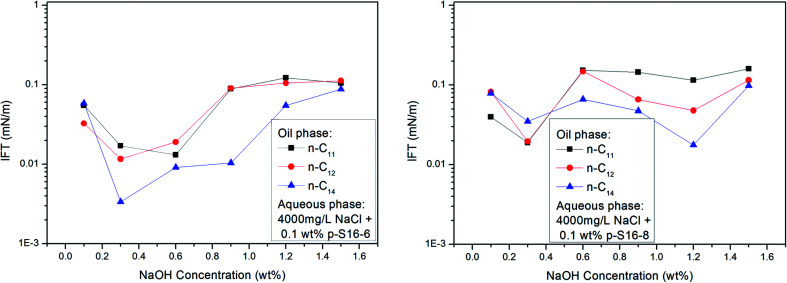
Effect of NaOH concentration on IFT of PDABS isomers.

With the shift of the benzene ring (hydrophilic group) to the center of the long alkyl chain (lipophilic group), the hydrophilicity of PDABS increases, its solubility in water increases, and its solubility in oil decreases; thus the effect of NaOH on the distribution of PDABS at oil/water interfaces should also be enhanced. The IFT values of *p*-S16-6 and *p*-S16-8 with the benzene ring closer to the center of the long alkyl chain are listed in Fig. 7. By comparison with *p*-S16-5 (see Fig. 6), it can be seen that NaOH plays an increasingly important role with the movement of the benzene ring towards the alkyl chain center. The trend is for IFT to become closer to that of the water-soluble *p*-S12-5.

It can also be seen from Fig. 6 that the *p*-S14-5, *p*-S16-5 and *p*-S18-5 homologues can display ULIFT (∼10^−3^ mN M^−1^) in a certain range of NaOH concentration.

### 3.3. Effect of NaOH concentration on interfacial tension when using crude oil as oil phase

**Fig. 8:**
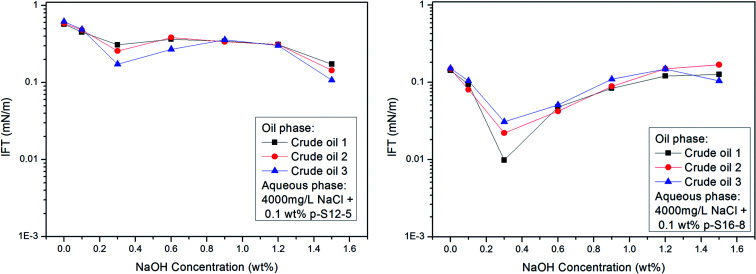
Effects of NaOH concentration on IFT of crude oil/water.

As already mentioned above, the acidic components in crude oil may react with NaOH to *in situ* generate active substances, which, by a synergistic effect with ABS, can effectively lower the IFT of the oil/water phase. We chose three crude oils with different EACN values (about 11, 12, and 14 respectively) as oil phases to study the effect of NaOH concentration on IFT between different crude oils and PDABS aqueous solutions. The results are shown in Fig. 8.

The effect of NaOH concentration on the IFT of crude oil/water is basically consistent with the variation trend of the IFT of a normal paraffin/water system, and no lower IFT is produced than that of normal paraffin. This shows that NaOH has a similar effect on crude oil as on normal alkanes. Even if the acidic or other components present in crude oil react with NaOH to *in situ* form active species, however, it is not obvious that this kind of active species will reduce the IFT of oil and water. Moreover, the composition of crude oil is complex and changeable; both the contents of acidic components in crude oil and active species generated after reaction, as well as the synergetic effect between “active species” formed *in situ* and ABS, need to be questioned.

### 3.4. Effect of ionic strength on interfacial tension

**Fig. 9:**
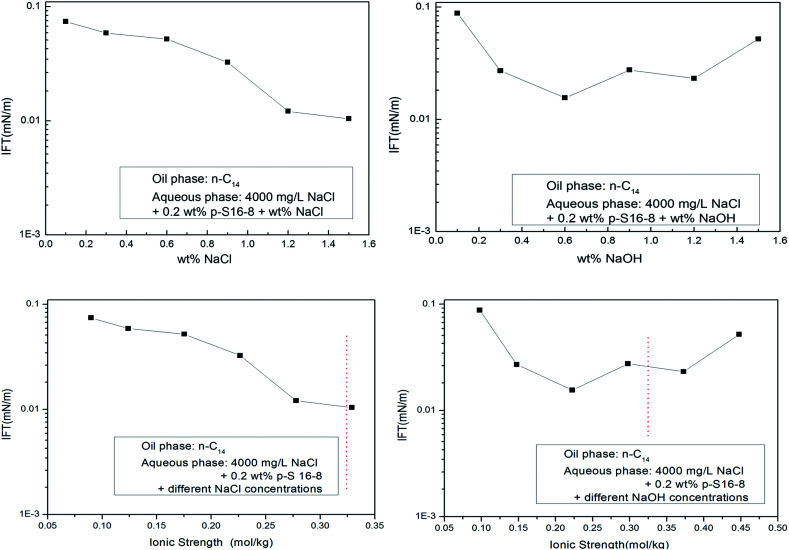
Effects of NaOH and NaCl concentration and ionic strength on IFT of normal alkane/water.

The NaOH influences the electrical double layer structure and the electrostatic effect of ABS in aqueous solution, leading to a change in the interfacial concentration of ABS, which ultimately affects the IFT. We roughly compared the effects of different electrolyte concentrations and ionic strengths on IFT for normal paraffin as oil phase (the ionic strength is half of the sum of the square of the molality of each ion in the solution, where each molality is multiplied by the valence number of the ion). The results are shown in Fig. 9.

We can see from Fig. 9 that with the increase of NaCl and NaOH concentration in aqueous solution, the IFT decreases. After simply converting both concentrations to ionic strengths, the trends remain similar in the same range of ionic strength (see the dotted red line). Despite the obvious simplifications assumed in this transformation, the concentrations and ionic strength have roughly similar effects on the IFT trend. Moreover, it is remarkable that the minimum IFT in NaCl aqueous solution is lower than that in NaOH aqueous solution. This also indirectly shows that NaOH cannot specifically interact with normal paraffins, and the interfacial properties have not been improved by using NaOH rather than NaCl in the aqueous phase.

### 3.5. The mechanism for the effect of NaOH concentration on interfacial tension

The roles of NaOH in the change of IFT are summarized below:

Firstly, simple consideration of the polarity characteristics of the water phase suggests that NaOH can increase the ionic strength of the water phase, which results in compression of the electrical double layer of hydrophilic groups. Due to the decrease in the area occupied by the molecules at the interface the interfacial concentration of surfactant increases; hence, there is a consequent increase in the IFT.

Secondly, with the continuous increase of the NaOH concentration, the electrical double layer is further compressed; the electrostatic interactions between hydrophilic groups are weakened and the ranges of electrical effects are shortened. Therefore, the water molecules can incorporate into the interface through the gap between loose polar groups. This is not conducive to a decrease in IFT, but to its increase.

Surfactant molecules can be driven into the oil phase by the salting out effect at a higher NaOH concentration. This improves the mutual attraction of lipophilic chains and the close-packed lipophilic groups at the interface. Thus, it makes the IFT decrease for a water-soluble surfactant. However, for an oil-soluble surfactant, the electrolytic concentration has little effect on the close-packing of longer chains, due to the inherently greater van der Waals attraction between longer chains. Therefore, a decrease of IFT no longer occurs at higher NaOH concentration for an oil-soluble surfactant.

**Fig. 10:**
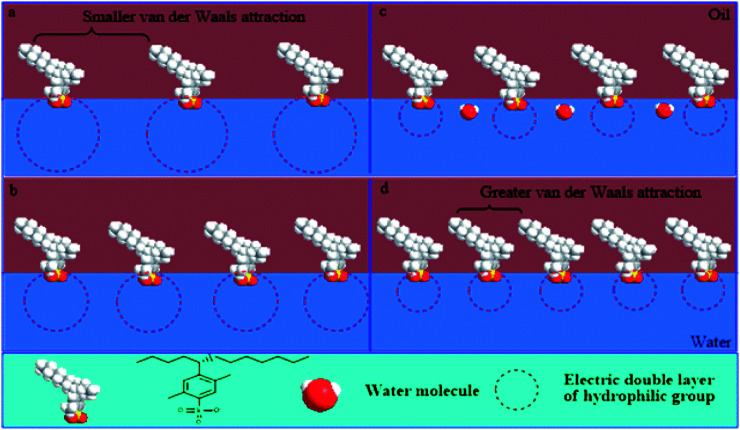
Effects of NaOH concentration on interfacial properties and arrangement at interface for water-soluble alkyl benzene sulfonates (the concentration of NaOH gradually increases in the order of a, b, c and d).

The effects of NaOH concentration on the electrical double layer of polar groups and the interfacial arrangement of a water-soluble surfactant are shown in Fig. 10.

## 4. Conclusions

For a system of normal paraffin and PDABS aqueous solution, the use of NaOH as an electrolyte changes the ionic strength of the aqueous phase, and thus changes the size of the electrical double layer of PDABS polar groups, to in turn influence the IFT. The increase of NaOH concentration results in compression of the electrical double layer (*i.e.*, a decrease in the area occupied by the molecules at the interface), which is beneficial to increasing the interfacial concentration of PDABS, such that IFT decreases gradually. However, with the NaOH concentration rising, water molecules can incorporate into the interface through loose hydrophilic groups, resulting in an increasing trend of IFT with the increase in NaOH concentration.

For a water-soluble PDABS, the “salting out” effect leads to the transportation of PDABS molecules into the oil phase at a higher NaOH concentration. The effective distribution of PDABS in the oil phase improves the mutual attraction of lipophilic chains and the close-packed lipophilic groups at the interface, thus again decreasing the IFT. For an oil-soluble PDABS, the increase in NaOH concentration has little effect on the distribution of PDABS in the oil phase, thus, a decrease of IFT no longer occurs at higher NaOH concentration.

The results showed that, in a certain range of NaOH concentrations, *p*-S14-5, *p*-S16-5 and *p*-S18-5 produced ULIFT. NaOH was roughly similar to NaCl in aqueous solutions, in terms of its effect on interfacial concentration and arrangement of PDABS surfactant molecules at the interface. The *n*_min_ values increase with increasing NaCl and NaOH concentration. The IFT decreases with increasing NaCl concentration, but for NaOH, the IFT increases again at the concentration of 0.9 wt% NaOH.

The effect of NaOH concentration on the IFT of crude oil is similar to that on the IFT of normal alkanes. There is no evidence to support the notion that active species can be formed by *in situ* reaction of NaOH with acidic or other components in crude oil, or that such active species have an obviously synergistic effect with PDABS on reducing the IFT between oil and water.

## Conflicts of interest

There are no conflicts to declare.

## Supplementary Material

## References

[cit1] Vonnegut B. (1942). Rev. Sci. Instrum..

[cit2] Rosenthal D. K. (1962). J. Fluid Mech..

[cit3] Torza S. (1975). Rev. Sci. Instrum..

[cit4] Chan K. S., Shah D. O. (1980). J. Dispersion Sci. Technol..

[cit5] Doe P. H., Wade W. H., Schechter R. S. (1977). J. Colloid Interface Sci..

[cit6] Zhao Y., Xu Z. G., Li Z. S., Qiao W. H., Cheng L. B. (2006). Pet. Sci. Technol..

[cit7] Zhang Y., Zhang G. Y., Wang P. W., Niu J. P., Guan J. C., Gu H. X. (2005). Acta Phys.–Chim. Sin..

[cit8] Cao Y., Zhao R. H., Zhang L., Xu Z. C., Jin Z. Q., Luo L., Zhang L., Zhao S. (2012). Energy Fuels.

[cit9] Zhao Y., Dong Y. H., Bi C. F., Fan Y. H. (2008). Acta Chim. Sin..

[cit10] Lashkarbolooki M., Riazi M., Ayatollahi S. (2016). Chem. Eng. Res. Des..

[cit11] Tichelkamp T., Teigen E., Nourani M., Oye G. (2015). Chem. Eng. Sci..

[cit12] Yuan F. Q., Cheng Y. Q., Wang H. Y., Xu Z. C., Zhang L., Zhang L., Zhao S. (2015). Colloids Surf., A.

[cit13] Li H. R., Li Z. Q., Song X. W., Li C. B., Guo L. L., Zhang L., Zhang L., Zhao S. (2015). Energy Fuels.

[cit14] Doe P. H., El-Emary M., Wade W. H., Schechter R. S. (1977). J. Am. Oil Chem. Soc..

[cit15] Doe P. H., El-Emary M., Wade W. H., Schechter R. S. (1978). J. Am. Oil Chem. Soc..

[cit16] Doe P. H., El-Emary M., Wade W. H., Schechter R. S. (1978). J. Am. Oil Chem. Soc..

[cit17] Zhu Y. W., Zhao R. H., Jin Z. Q., Zhang L., Zhang L., Luo L., Zhao S. (2013). Energy Fuels.

[cit18] Zhang L., Luo L., Zhao S., Xu Z. C., An J. Y., Yu J. Y. (2004). J. Pet. Sci. Eng..

[cit19] Lashkarbolooki M., Ayatollahi S. (2016). Fluid Phase Equilib..

[cit20] Lashkarbolooki M., Riazi M., Ayatollahi S., Hezave A. Z. (2016). Fuel.

[cit21] Zhao R. H., Zhang L., Zhang L., Zhao S., Yu J. Y. (2010). Energy Fuels.

[cit22] Zhao R. H., Huang H. Y., Wang H. Y., Zhang J. C., Zhang L., Zhang L., Zhao S. (2013). J. Dispersion Sci. Technol..

[cit23] Mosayebi A., Angaji M. T., Bourbour K. (2016). Energy Sources, Part A.

[cit24] Chen Y. M., Wang H. H., Yu J. Y. (2001). Acta Pet. Sin..

[cit25] Chu Y. P., Luo L., Zhang L., Wang L., Zhao S., Yu J. Y. (2004). Acta Phys.–Chim. Sin..

[cit26] Chu Y. P., Gong Y., Tan X. L., Zhang L., Zhao S., An J. Y., Yu J. Y. (2004). J. Colloid Interface Sci..

[cit27] Cayias J. L., Schechter R. S., Wade W. H. (1976). Soc. Pet. Eng. J..

[cit28] Cash L., Cayias J. L., Fournier G., Macallister D., Schares T., Schechter R. S., Wade W. H. (1977). J. Colloid Interface Sci..

[cit29] Zhang L., Luo L., Zhao S., Yu J. Y. (2002). J. Colloid Interface Sci..

[cit30] Chu Y. P., Luo L., Chen G. Y., Fu X. S., Zhang L., Wang L., Zhao S., Yu J. Y. (2007). J. Dispersion Sci. Technol..

[cit31] Miller C. A., Scriven L. E. (1970). J. Colloid Interface Sci..

